# Table Olives: An Overview on Effects of Processing on Nutritional and Sensory Quality

**DOI:** 10.3390/foods9040514

**Published:** 2020-04-20

**Authors:** Paola Conte, Costantino Fadda, Alessandra Del Caro, Pietro Paolo Urgeghe, Antonio Piga

**Affiliations:** Dipartimento di Agraria, Università degli Studi di Sassari, Viale Italia 39/A, 07100 Sassari, Italy; pconte@uniss.it (P.C.); cfadda@uniss.it (C.F.); delcaro@uniss.it (A.D.C.); paolou@uniss.it (P.P.U.)

**Keywords:** composition, nutritional properties, polyphenols, sensory analysis, table olives

## Abstract

Table olives are a pickled food product obtained by a partial/total debittering and subsequent fermentation of drupes. Their peculiar sensory properties have led to a their widespread use, especially in Europe, as an appetizer or an ingredient for culinary use. The most relevant literature of the last twenty years has been analyzed in this review with the aim of giving an up-to-date overview of the processing and storage effects on the nutritional and sensory properties of table olives. Analysis of the literature has revealed that the nutritional properties of table olives are mainly influenced by the processing method used, even if preharvest-factors such as irrigation and fruit ripening stage may have a certain weight. Data revealed that the nutritional value of table olives depends mostly on the balanced profile of polyunsaturated and monounsaturated fatty acids and the contents of health-promoting phenolic compounds, which are best retained in natural table olives. Studies on the use of low salt brines and of selected starter cultures have shown the possibility of producing table olives with an improved nutritional profile. Sensory characteristics are mostly process-dependent, and a relevant contribute is achieved by starters, not only for reducing the bitterness of fruits, but also for imparting new and typical taste to table olives. Findings reported in this review confirm, in conclusion, that table olives surely constitute an important food source for their balanced nutritional profile and unique sensory characteristics.

## 1. Introduction

The olive (*Olea europaea* L.) originates in the Mediterranean countries; it can be found in the wild form in the Middle East and it is widely distributed around the world, especially in the Mediterranean region, where about 96% of the world’s production of olives occurs [1]. It grows in form of an evergreen tree, and the first domestic cultivation dates to the Minoan period (3500–1500 BC) in Crete [2]. The fruits are mainly used to produce oil and table olives, a widely consumed food of the Mediterranean countries. The World Catalogue of Olive Cultivars [3] reports about 2500 olive varieties, but only 10% of them can be considered commercial, and their selected use (oil, table or both) is determined by different parameters. Table olives, in fact, are prepared from varieties low in oil content, medium to large in size and appropriate in shape, with flesh-to-pit ratios higher than 4, green to black skin and appropriate texture (depending on the skin color). The main table olive varieties used in the five major producing countries are Gordal, Manzanilla and Hojiblanca for Spain; Aggezi Shami, Hamed and Toffahi for Egypt; Gemlik, Memecik and Memely for Turkey; Konservolia, Chalkidiki and Kalamon for Greece; Azeraj and Sigoise for Algeria. The International Olive Oil Council has estimated for the 2017/2018 crop year that Egypt, with 655.000 tons, will be for the first time the world leading country for table olive production. The olive trees produce drupes that are each constituted by a thin epidermis and a soft mesocarp surrounding a stone containing the seed [4]. The epidermis (1.5–3% of the total weight) has a protective function against external attacks and it is mainly constituted of cellulose and cutin [5,6]. Olive mesocarp represents 70–90% of the weight. The stone accounts for the 10–30%, while the seed is about 1–3% of the whole fruit, and it is made up mainly of lipids [7]. Olives fruits have a round to ovoid shape, and their weight ranges from 0.5 to 20g, with a major frequency in the weight class of 3–10 g. Additionally, they are characterized by a strong bitter taste that decreases with fruit ripening, during which the peel color changes from green to light-yellow, purple-red and purple-black. The principal components of olives are water (60–75%), lipids (10–25%), reducing sugars (2–5%) and phenolic substances (1–3%) [4,8]. Olives, moreover, have good amounts of tocopherols, carotenoids [9] and minerals [10]. Among the cited components, olives are very rich in polyphenols, which are important for the sensory properties of olives, and may have various health promoting activities [11]. Polyphenols in olives belong to the following five different classes [12,13]: acids (caffeic, gallic, syringic); alcohols (tyrosol, hydroxytyrosol); flavonoids (luteolin-7-glucoside, cyanidin-3-glucoside); secoiridoids, such as the bitter oleuropein that diminishes during maturation, demethyloleuropein and the dialdehydic form of elenolic acid linked to tyrosol and hydroxytyrosol—whose amount in contrast, increases with fruit maturation; and lignans (1-acetoxypinoresinol, pinoresinol). The International Olive Oil Council (IOC) [14] has recently reported on the importance of table olives in an every-day diet, as this specialty is the most consumed fermented food in Europe and accounts for a worldwide production of close to 3 million tons. Some authors recommend daily consumption of a serving size [15,16].

Tables olive processing involves the removal of the bitter taste, and in most cases the subsequent fermentation that imparts to the fruits a well-defined sensory profile, while avoiding the growth of pathogenic bacteria and giving proper stability [4]. Unit operations involved during processing and storage, on the other hand, may have important effects on the nutritional and sensory characteristics of fresh olives, and this review has the aim of giving an up-to-date overview of how the processing and storage of table olives may affect the nutritional and sensory characteristics of this pickled food.

## 2. How Processing Influences the Nutritional Properties of Table Olives 

**Figure 1:**
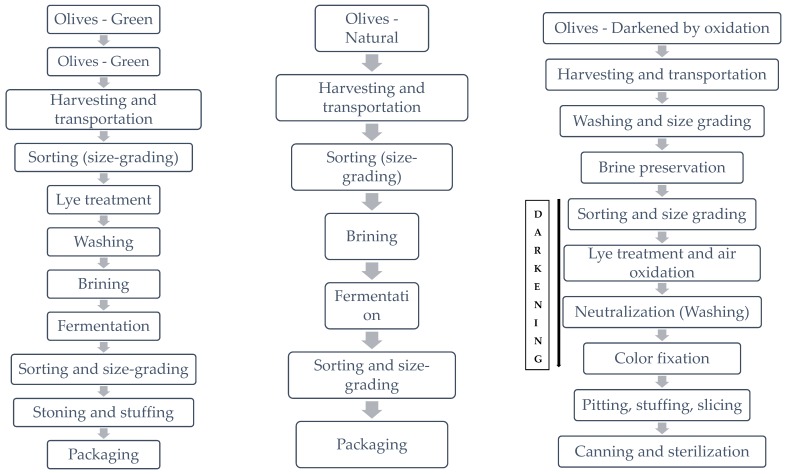
Flow sheets of processing of treated green, natural and darkened by oxidation olives.

According to IOC [17] “Table olives are the product prepared from the sound fruits of varieties of the cultivated olive tree that are chosen for their production of olives whose volume, shape, flesh-to-stone ratio, fine flesh, taste, firmness and ease of detachment from the stone make them particularly suitable for processing; treated to remove its bitterness and preserved by natural fermentation, or by heat treatment with or without the addition of preservatives; packed with or without covering liquid”. Classification of table olives could be made on the basis of the ripening stage at harvest (green, turning color and black), trade preparations (treated, natural, darkened by oxidation, dehydrated and/or shriveled, specialties) and styles (whole, pitted, stuffed, salad and other). According to the trade preparations, about 80% of the world’s production is covered by three commercial processing methods: treated green olives (or Spanish style green olives); olives darkened by oxidation (ripe olives) (Californian style); natural (mainly black) olives (Greek style) (Figure 1). Processing, in any case, promotes a quantitative and qualitative evolution in the phenolic compounds of table olives, thereby changing their sensory and health properties [18]. 

In the following pages, we will review the effects of the trade preparation methods and styles, and the influence of the microbial starter on the table olives’ nutritional quality.

The following databases were used for the bibliographic research: Web of Science (2000–2020), Scopus (2000–2020) and Food Science and Technology Abstracts (2000–2020). Some papers deal with either nutritional or sensory topic; thus, we discuss them in both sections. Results are summarized in Table 1.

### 2.1. Trade Preparations

#### 2.1.1. Treated Green Olives or “Spanish Style”

Basically, lactic acid bacteria (LAB) ferment brined olives, which have been previously debittered through a chemical hydrolysis of oleuropein by a lye treatment in a 1.5–4.5% (w/v) NaOH solution until 2/3 of the mesocarp is interested; after that, olives are drained and washed with water. The alkali treatment speeds up the fermentation, as it increases the skin permeability and the efflux of fermentable compounds and nutrients in the sodium chloride (NaCl) brine. Diffused sugars are converted into lactic acid by LAB, which predominate after the first days of processing. The final product, which is obtained after 30–60 days from brining, has a pH of 3.8–4 and 5–6% NaCl, and it is shelf stable in its final pack. The use of sorbic acid or application of pasteurization have been reported to extend the shelf life [12]. Nutritional losses from the fresh olives result both from the alkali treatment and from leakage of soluble constituents from olive mesocarp to brine. Sakouhi et al. [19] studied how ripening and processing may change the contents of α-tocopherol and fatty acids (FA) of three Tunisian varieties (Meski, Picholine and Sayali). They harvested fruits while green but also at cherry and black stages of ripening, and showed that α-tocopherol increased during ripening and decreased after fermentation, especially when black olives were used. Data on fatty acids revealed that for the three cultivars, the ratio of polyunsaturated (PUFA) to saturated fatty acids (SFA) was lower and irregular at the cherry stage, with respect to the other two ripening stages, but this ratio increased after processing in Meski and Picholine olives at a value higher than 1.5, which is usually associated with health-promoting capacity [20]. Lanza et al. [21] focused on the nutritional properties of Spanish style Italian “Intosso d’Abruzzo” fermented olives. The authors showed that these olives may be considered a food with a high nutritional potential for their low and balanced fat profile, especially for the high rate between monounsaturated fatty acids (MUFA) and SFA; the appreciable amounts of polyphenols, α-tocopherol, minerals and fiber; an adequate content of essential amino acids; and a normal NaCl level. López-López et al. [22] verified whether the Spanish-style could affect the FA and triacylglycerol (TAG) composition of Manzanilla ad Hojiblanca olive cultivars. The authors used principal component analysis (PCA) to analyze data, and found that FA, TAG and nutritional fat subclasses were influenced mainly by cultivar and to a very low extent by processing, and that the ratio PUFA/SFA was slightly lower than the 0.5 that is recommended for prevention of coronary hearth diseases. Cano-Lamadrid et al. [23] evaluated the influences of three different irrigation regimes, from normal to moderate stress, on the fatty acid composition of green Manzanilla olives. Results showed that olives grown under moderate irrigation stress had the highest content of PUFA, and in particular, linoleic acid. The same research group [24] carried out a study by applying a similar irrigation experimental plan and evaluated its influence on the phenolic profile of Spanish style Manzanilla olives. Results evidenced that a moderate level of irrigation increased the amounts of some polyphenols in table olives, especially those of compounds with health promoting activities, such oleuropein and oleoside diglucoside. Mastralexi et al. [25] followed the evolution of hydrophilic and hydrophobic antioxidants of the protected denomination of origin (PDO) “Prasines Elies Chalkidikis” olives prepared at industrial scale and following a storage period of 12 months. The authors found that NaOH debittering and subsequent washing reduced the total polyphenol content by at least 2/3. Oleuropein was completely removed by the alkali treatment, and only hydroxytyrosol, tyrosol and oleoside-11-methyl ester were found at the end of washing in significantly higher concentrations with respect to fresh olives. The hydrophobic nutrients α-tocopherol and squalene were not affected by processing. The ensuing storage in brine led to a decrease of squalene and phenols; the latter, however, were high enough to permit to use the health claim on olive oil polyphenols [26]; however, the authors highlighted some concerns about an overly high final salt content. 

Results of the papers above-discussed reveal that treated green olives may have an important nutritional profile, regarding the adequate FA content, and the appropriate PUFA/SFA ratio and α-tocopherol minerals. Polyphenols, on the other hand, despite undergoing a severe loss due to the lye treatment, remain at a good level. 

#### 2.1.2. Natural Olives 

This trade preparation is performed by harvesting olives at the three ripening stages and then fermenting them directly in brine. Aids may be used to further preserve olives. Primarily, fruits at the black stage are used, and this preparation is known as “Greek style”. The fermentation may be achieved in an 8–10% NaCl brine in anaerobic or aerobic conditions. In the last case, a modification of the fermenter is obtained by bubbling air through a central column. The regulation of NaCl in brine drives the type of fermentation, because when NaCl is higher than 8% yeast (Y) predominates, while an NaCl concentration of 3–6% may promote the LAB growth in turning or black olives. The anaerobic fermentation requires a long time, from 8 to 12 months, to solubilize oleuropein in the brine, while the aerobic system significantly reduces the process time and limits gas-pocket spoilage and shriveling of fruits [27]. The obtained olives may be packed directly in brine and sold, or they may also be submitted to pasteurization or even preserved with the addition of sorbic acid at 0.5 % to the packing brine. The nutritional loss is mainly caused by leakage of soluble compounds into the brine during fermentation and storage. Boskou et al. [28] worked on five different commercial samples of black Greek-style fermented olives: he analyzed the polyphenolic pool. He identified 13 different polyphenols; the main ones were hydroxytyrosol, oleanolic acid and tyrosol. Data obtained on the different samples evidenced, regardless of the cultivar and preparation, an appropriate amount of polyphenols for covering the requested daily intake, which could be satisfied by 5 or 10–12 olives in North European countries or Greece, respectively. Similar results were obtained by Pires-Cabral et al. [29] on fermented table olives belonging to three Portuguese cultivars (Cobrançosa, Galega and Maçanilha Algarvia). Results showed an important dietary fiber and polyphenol content and high amounts of PUFA in all samples, with particular emphasis on the Maçanilha cultivar, which was able to provide 13.1% of the recommended daily intake of PUFA. In a further paper of Pires-Cabral et al. [30] the authors studied the nutritional properties of a Portuguese olive cultivar fermented in a reduced NaCl brine (4% NaCl + 4% KCl). The authors highlighted the use of this technology in halving the Na content of olives and in increasing by six and four-times, K and Ca, respectively, in comparison to samples fermented in a conventional brine (8% NaCl). D’Antuono et al. [31] revealed by liquid chromatography–mass spectrometry/mass spectrometry (LC-MS/MS), for the first time in Greek-style processed olives, the presence of three nutritionally important polyphenols—hydroxytyrosol acetate (HTAc), caffeoyl-6′-secologanoside (SEC) and comselogoside (COM); the first was previously found only in the olive oil of table olives [32], and the other two in air-dried olives [33]. The authors also showed the good bioaccessibility of these phenolic compounds and evidenced that these table olives can be considered a functional food. In a more recent paper of Fernández-Poyatos et al. [34], a complete characterization of the polyphenolic and inorganic fractions of Cornezuelo natural processed table olives was carried out. The authors identified thirty phenolic compounds, the most representative being oleuropeine and comselogoside isomers, and a high amount of Ca. The authors submitted the polyphenolic extract to a simulated gastrointestinal digestion and found that, although almost 50% of it has been digested, an important residual in vitro antioxidant activity remained. In a study of Rodríguez et al. [35], thirty-two commercial samples obtained with ten cultivars and different styles were analyzed for phenol composition, with emphasis on the nutritional important compound 3,4-dihydroxyphenylglycol (DHPG) [36]. Data obtained on natural-style olives processed at the black stage revealed a high concentration of DHPG; thus, these samples have an interesting nutritional potential.

Direct brine fermentation confirms, thus, the disadvantage, with respect to treated olives’ trade preparation, of longer processing times, but it results in olives that contain health-promoting polyphenols.

#### 2.1.3. Dehydrated and/or Shriveled Olives 

According to IOC [17], this trade preparation is carried out on “green olives, olives turning color or black olives that have undergone or not to mild alkaline treatment, preserved in brine or partially dehydrated in dry salt and/or by heating or by any other technological process”. 

Drying is one of the oldest unit operations for food stabilization, and it is based on constitutive water removal under water activity (a_w_) values below the threshold for microbial growth. Drying of foods like olives is often carried out using cabinet drying equipment. Nutritional loss is expected due to thermal damage. Mantzouridou et al. [33] evaluated the influences of mild drying conditions and storage for 6 months at 4 or 20 °C in an air, nitrogen or vacuum atmosphere on the phenol composition of intermediate moisture olives (a_w_ =0.89). After drying, the authors found a significant decrease of the single polyphenols, up to 73%, that continued during storage. They also evidenced that the best combination in reducing such a loss during storage was keeping olives under vacuum at 20 °C, which assured the highest contents of nutritionally important polyphenols, such as oleuropeine and hydroxytyrosol. Other important results were the high contents of eleanolic acid and elanolic acid glicoside, which are hydrolytic oleuropein derivatives and may be considered bioactive compounds. The authors concluded that, despite this loss, the olives maintain a sufficient polyphenol content to assure a proper shelf life. In another paper, Lanza et al. [37] evaluated the nutritional properties of oven-dried Ferrandina table olives. Although the authors found a low protein content, they observed an important contribution from some essential amino acids. They also revealed that the fat content was high, but with a balanced composition of PUFA. Moreover, the dried fruits contained appreciable amounts of phenols and tocopherols.

The expected nutritional loss due to the thermal treatment has been demonstrated by the above-cited papers, which, however, highlighted adequate contents, in the finished products, of some specific compounds, such as essential amino acids, fats, polyphenols and tocopherols. 

#### 2.1.4. Other Processing Methods and Stabilization Treatments

The “alcaparras” are a Portuguese table olive specialty. Alcaparras are prepared with olives harvested at the green or yellow-green stage. Fruits are cut with a hammer to separate pulp from stone and then halved; thus, they may be classified as “stoned halved olives” [17]. After that, halved fruits are dipped in water several times over a week, in order to remove oleuropein by diffusion, and this results in a significant loss of all polyphenols [58]. Stabilization of olives is carried out by placing them in brine. Sousa et al. [50] did a complete nutritional characterization of thirty stoned alcaparras table olive samples along three production seasons. The caloric value of processed olives is lower with respect to the majority of other commercial samples; moreover, they have a higher content of oleic acid and a lower content of α-tocopherol, if compared to olives prepared in other styles, although the authors evidenced that a serving size may provide a moderate contribution to the daily intake of tocopherols. Similar results were obtained by a further study of the same research group that investigated the effects of the influence of cultivar on main nutritional quality of alcaparras olives [51]. 

Another diffused style in the major producing countries is the “cracked olives style,” which is when the “whole olives [are] subjected to a process whereby the flesh is opened without breaking the stone (pit), which remains whole and intact inside the fruit” [17]. Moreno-Baquero et al. [53] substituted up to 50% of NaCl in packing brines with combinations of CaCl_2,_ KCl and NaCl, and checked for the influence on the mineral nutrients of cracked Aloreña olives, one of the three processing styles of this fruit (a complete description will be reported in Section 3.1.4). The authors found that the reduction of NaCl resulted in significant reduction of flesh Na content, with respect to the traditional packed product; moreover, the contents of K and Ca increased. An important contribution to the knowledge of polyphenol changes during table olive processing was given by Mousori et al. [38], who used nuclear magnetic resonance (NMR) to detect new compounds and relative metabolites from Megaritiki table olives and wastewaters. The authors detected, for the first time, compounds that are unique for the species (rengyoxide and cleroindicin C) and for table olives (haleridone), and found four lactones derived from oleuropein hydrolysis. Another promising non-chemical debittering unit operation has been proposed by Habibi et al. [59], who checked the influence of ultrasound on the nutritional content (protein, ash and fat) of natural fermented table olives. Ultrasound-assisted debittering (UAD) was carried out both in water and in brine, and two unassisted controls were considered. The UAD significantly decreased the debittering time and left unchanged all the nutritional parameters, with respect to the controls, except for ashes that increased in UAD samples. Saúde et al. [54] tested the effect of brine NaCl replacement with CaCl_2_ and/or KCl on nutritional properties of cracked Maçanilha Algarvia table olives. The authors evidenced that the combination of 4%NaCl+4%KCl—that was the only one sensorially accepted—resulted in samples with lower fat contents; similar dietary fiber contents, phenolic compound contents and Ca contents with respect to the control (8% NaCl); and increased K and reduced Na, thereby improving the nutritional quality of the obtained reduced salt olives.

With the aim of substitute traditional thermal stabilization technologies, the use of high hydrostatic pressure (HHP) was proposed on Greek-style Turkish table olives [39]. HHP assures both microbial elimination and no heat damage; thus, nutritional characteristics may be maintained [60]. The authors showed that the HHP treatment, aside from stabilizing the product, resulted in an increase up to 2.1–2.5-fold of total phenolics, and in particular, of hydroxytirosol, probably because the HPP treatment allows a higher extraction rate. The conclusion was that this unit operation can be proposed as an alternative to the traditional heat treatments. 

Results obtained using new unit operations such as UAD and HPP or reducing brine salt content have, thus, proved to be beneficial to improving the nutritional power of table olives. 

#### 2.1.5. Comparison among Different Trade Preparations and Styles

Several papers focused comparisons of the three main trade preparations—treated olives, natural olives and olives darkened by oxidation—on the nutritional quality of olives, and on other styles. 

In the very comprehensive paper of Romero et al. [32], the effects of the above-cited main trade preparations and of cultivars on the single polyphenolic compounds extracted from aqueous and lipid phases of table olives were studied. Concerning the aqueous phase, the authors showed the highest amounts of polyphenols in turning color olives, as they are submitted only to two dilution operations, while ripe oxidized olives had the lowest content. The advanced degree of ripeness of black olives processed with the Greek-style resulted in the highest anthocyanin content. The analysis of the lipidic phase, therein carried out for the first time, gave very important knowledge, as the authors evidenced a unique phenolic profile that is different from that of raw fruits. In fact, aglycons of oleuropein and ligustroside were absent in table olives, which, instead, presented for the first time the compound catechol. The authors concluded that table olives are a rich source of antioxidants, in some cases even more than virgin olive oils. 

In three papers of López-López et al. [52,61,62], an extensive analysis of FA composition, provitamin A carotenoids and Vitamin B was carried out on 67 commercial samples of table olives prepared according to the three above-cited preparations and with different cultivars and styles. Data presented by the authors showed which preparation-styles had the better nutritional profiles. In particular, the authors showed that there is a large variability in carotenoids content and that this is mainly due to the cultivar used [61], while they demonstrated that is possible to discriminate cultivars and commercial preparations by statistically analyzing the fat profiles with a discriminant analysis [52] and that the best trade preparation to maintain the Vitamin B content is the natural style, followed by the treated olive style [62]. Romero et al. [56] made for the first time an important study on triterpenic acids—which have been reported to have an anti-cancer activity [63]—of seventeen different olive cultivars processed according to the Spanish, Greek and Californian styles. The authors evidenced that placing olives directly in brine resulted in a very high content of triterpenic acids, with respect to the alkaline treatment of Spanish style. Additionally, the authors found that natural black olives have a much higher content of these bioactive compounds than olive oil, thereby concluding that table olives should be nutritionally reevaluated. Jiménez et al. [57] tested the effects of cultivar, processing type (darkening by oxidation, brine fermentation, or drying by oven or salt), and the ensuing storage on the fat and dietary fiber of six table olive cultivars, with emphasis on some properties of dietary fiber. The authors reported that obtained olives had a high content of fiber, but also that the water holding capacity of the alcohol insoluble residue is like that reported for other vegetables. Ben Othman et al. [40] studied the total polyphenol contents, the single polyphenols and the antioxidant capacities of four Tunisian table olives (Meski, Chemlali, Besbessi and Tounsi); one of them was harvested at four different ripening stages and processed with the natural style or dry-salting. The authors detected 14 different phenolic compounds, mostly hydroxytyrosol and tyrosol, while oleuropein was not detected. Results obtained for total phenol content and antioxidant activity values encouraged the authors to conclude that studied samples had an important amount of antioxidant compounds. Valenčič et al. [41] compared the effects of two processing methods, the traditional regional and modified Spanish style, and of storage (60 and 180 days), on the phenol contents of two Slovenian table olives. The traditional method involves debittering olives in water for 10 days followed by fermentation in brines at increasing NaCl concentration. The authors found a significantly higher biophenol content in the olives processed with the traditional method, and this resulted in the inhibition of LAB growth. Zoidou et al. [42] made a very comprehensive study on several commercial samples of Greek table olives (nine different cultivars and five processing-styles) to find which of them possessed the highest concentration of the nutritionally important phenolics oleuropein and hydroxytyrosol. The authors found that the dry-salting process of the Throuba Thassos olives allows obtaining olives with a very high oleuropein content. 

Lanza et al. [64] studied the effects of two modified Greek methods of preparation on the nutritional quality of each of two Italian table olive cultivars, Itrana and Oliva bianca di Itri. The first method implies a first water immersion step, followed, after 15–45 days, by NaCl addition to obtain an 8% brine. The second one was carried out by placing olives directly in brine prepared with a double-salting procedure that consists of adding half of the NaCl at the beginning and the other half after 15 days of brining. The authors found that the best method was the double-salting, as both olive cultivars have an appreciable amount of fiber and polyphenols, and Itrana cultivar has a PUFA/SFA ratio of about 0.4–0.5; that is the value recommended by nutritional guidelines [65]. Savas et al. [55] used different debittering methods in relation to the nutritional properties of Turkish Domat cultivar table olives. The methods were the following: lye at 1% NaOH, immersion in tap water and scratching followed by CaCl_2_ immersion and different brining replacements with reduced-salt brines. The best preparation was the scratching method in low-salt brines, as it resulted in table olives with reduced salt contents. Melliou et al. [43] set up an advanced HPLC-MS fast method to detect single polyphenols in darkened by oxidation (Manzanilla) and dry-salted (Mission and Throuba Thassos) table olives. The authors confirmed the results of Zoidou et al. [42], which showed that dry salting is the best processing style for retaining polyphenols in table olives.

Comparisons of the main trade preparations and other styles seem to confirm that the best nutritional table olive profile could be achieved by placing olives directly in brine.

### 2.2. Influence of Starters

The use of LAB or Y starter cultures could notably improve the fermentation of table olives, as the process may be shortened due to a rapid decrease of the bitter oleuropein trough hydrolysis mediated by the microbial enzymatic activity. These starter cultures should grow rapidly and predominate starting from refrigerated temperatures; they have a homofermentative metabolism and tolerate NaCl and glucosidated polyphenols and inhibit foodborne pathogens [66]. The above-cited characteristics are rarely found contemporarily in starter cultures, and this results in the scarce success of commercially available starter cultures, because the strains have not been adequately adapted for this use [67]. More success could surely come from starter cultures isolated from the olive fruits and brine. Recent studies highlighted the potential of starter cultures to improve the nutritional profiles of table olives. Servili et al. [44] evidenced the role of a *Lactobacillus pentosus* strain in polyphenol release from flesh to brine. The authors used appropriate scanning electron microscopy (SEM) to follow the microstructural changes of cells and tissues during the brining process, and found that skin cuticle tissues of LAB inoculated olives were totally altered, while normally fermented olive tissues were intact. The authors hypothesize that the skin degradation resulted in an increased permeability and diffusion of polyphenols from flesh to brine, thereby reducing the debittering time. Opposite results were found by Pistarino et al. [46], who showed that polyphenol loss from olives was enhanced by the process temperature and not by the use of LAB or LAB plus Y. Tataridou et al. [45] studied the effect of indigenous strains of *Lactobacillus plantarum* able to hydrolyze oleuropein on the phenol profile of green and black table olives placed in brines with low NaCl content. The authors found that the starter had an inhibitory effect on pathogen growth and that the obtained olives were significantly richer in phenols, especially hydroxytyrosol and tyrosol, and had lower NaCl content, with respect to an industrial product, thereby having a better nutritional profile. Durante et al. [47] used different starter cultures made up of a mixture of Y and LAB to assess changes in carotenoids, phenolics, triterpenic acids, vitamins, fatty acid profiles and antioxidant activity on black table olives from Italian and Greek cultivars prepared in the natural style. The authors, although they did not make any comparison with samples fermented with spontaneous microbiota, found that the obtained table olives were rich in monounsaturated fatty acids (MUFA), polyphenols, tocopherols and triterpenic acids, so that they may provide health benefits. D’Antuono et al. [48] evaluated the content of single polyphenols of natural table olives obtained with autochthonous LAB and Y starters and compared them with samples obtained by the market. The authors found that LAB+Y olives had quite always significantly higher polyphenol content than commercial samples, but no valid explanation was reported for this result. In particular, they found high contents of tyrosol and hydroxytyrosol that were up to eight times higher with respect to the virgin olive oils obtained by the same olives. Tufariello et al. [49] compared the effects of a previously selected *Saccharomyces cerevisiae* strain with a commercial preparation of the same Y and with a control without Y in terms of the nutritional properties of three olive cultivars prepared with the natural style at the black stage. The starters allowed a more rapid debittering process and permitted an increase in hydroxytyrosol, tyrosol and verbascoside on the olives, with respect to the control sample, thereby improving the nutritional value. 

The above-cited papers highlight the importance of autochthonous starters on table olive fermentation and leave the field open to additional research directed toward finding appropriate commercial starter cultures, at least for the main trade preparations.

## 3. How Processing Influences the Sensorial Quality

The conversion of fresh, inedible olive fruits to edible table olives involves, mainly, fruit debittering and the development of sensory characteristics (odor, taste and texture) that are unique to this food specialty. Researchers have given importance to this topic both to find the effects of unit operations and starters on sensory characteristics of table olives and to find appropriate sensorial profiles of selected trade preparations. Sensory analysis by trained assessors is generally carried out by quantitative descriptive analysis (QDA), unless differently indicated, using internationally recognized standards. Results will be summarized in Table 2.

### 3.1. Trade Preparations

#### 3.1.1. Treated Green Olives or “Spanish Style”

This trade preparation produces table olives in which the bitter taste is absent, while salty and acidic taste and other flavors derived from fermentation are present. 

González et al. [68] tried to find a correlation between sensory and objective results with the aim to find the best match between parameters. The QDA considered the descriptors acidity, bitterness, color, firmness, saltiness and intensity and persistency of nasal aroma. Several direct and inverse correlations were found between sensory descriptors and instrumental data, such as that between instrumental and subjective color and polyphenol content and fruit color; the best descriptors that characterized the table olives were color, firmness, acidity and saltiness. Marsilio et al. [69] did a sensory study (using Nocellara messinese olives at the green stage) on the influence of alkali neutralization with CO_2_, in comparison with traditional washing with water. The eight-member trained panel rated the appearances, colors, flavors (acid, bitter, salty), odors and textures (crispness and firmness) of processed olives [112]. The assessors judged olives treated with CO_2_ as more acidic than the control, while no differences were found for texture, although care should be taken to reduce the increase of the buffering potential of brines that can result in inadequate pH lowering of brines. Yilmaz et al. [70] carried out a sensory evaluation of different table olives and investigated the consumer preferences. The six-member panel used the descriptors appearance, aroma, flavor and texture of commercial green table olives of four Turkish cultivars [113]. A total of 50 people carried out the consumer test by using a scale from 0 to 9. Results evidenced that the sensory differences were cultivar dependent, and that, for consumer preference, the most important factor in willingness to buy was the mouth feeling. The effects of brines obtained with different NaCl concentrations on gustatory and kinesthetic sensations of treated green table olives were tested by Moreno-Baquero et al. [71]. The authors used fifteen different brines made up of NaCl (4–10%), KCl (0–4%) and CaCl_2_ (0–6%). A panel of nine trained assessors evaluated negative, gustatory and kinesthetic attributes [114]. Multivariate statistical analysis (MSA) was used to correlate the initial brine concentrations with sensory attributes. Saltiness was significantly related to NaCl and KCl levels, while bitterness, hardness, fibrousness and crunchiness were in relation to the CaCl_2_ percentage. The authors concluded that the models developed in the work can be useful in the production of particular table olives. Villegas Vergara et al. [115] proposed two different brine acidification methods—the first with CO_2_ gas, and the second by mixing LAB with lactic and hydrochloric acids—and evaluated their influence on the sensory properties of olives (cv. Conservolea). A ten-member panel carried out the sensory analysis [116]. The authors found that the acidification step is useful in helping the fermentation process and it has no effect on the sensory profile of olives. Bautista-Gallego et al. [117] used fermented Manzanilla olives to evaluate the influence of the addition of zinc chloride (ZnCl_2_ at 0.00%, 0.25%, 0.50%, 0.75% and 1.00%) to brine on increasing the olives’ shelf life and improving their sensory properties. A panel of twelve trained members used two protocols [81]. In the first one, the ranking test, 0.00 ZnCl_2_ was used as the control and panelists ranked the other samples by dissimilarity to the standard (1 more similar, 5 less similar). In the second protocol, the A–Not A, judges were asked to decide if samples were the same (sure or not sure) or different (sure or not sure). The two tests did not give significant differences between the control and the olives added with ZnCl_2_, thereby suggesting that this salt does not affect the sensory characteristics of the samples studied. The influence of the substitution of NaCl with KCl and CaCl_2_ on the sensory profile of Manzanilla olives was studied by López-López et al. [72]; they used 16 brines with different salt concentration ranges (40–100 g/L of NaCl, 0–60 g/L of Kcl and 0–60 g/L of CaCl_2_). Nine experienced assessors determined negative sensations and used the descriptors for taste and kinesthetic attributes. [116]. Data were statistically treated with partial least square analysis (PLS) and principal component analysis (PCA). The assessors found a decrease in saltiness and an increase in bitterness at increasing Ca amounts in the olive pulp. Data of Ca contents were highly correlated by PLS both with some kinesthetic (hardness, fibrousness, crunchiness) and taste attributes (bitterness and saltiness); PLS used Ca, K and Na pulp content to estimate sensory characteristics of samples. The influences of three different irrigation regimes, from normal to moderate stress, on sensory properties of green fermented Manzanilla olives, were evaluated by Cano-Lamadrid et al. [73]. Eight trained panelists evaluated attributes related to main sensory attributes of flavor and texture [116]. A consumer acceptability test with a nine-point scale was also carried out by 100 assessors. Olives grown under soft stress conditions were rated as the best for the more important descriptors, and they were preferred among Spanish consumers. Results confirmed those obtained in a previous work [23]. López-López et al. [74] developed a sensory profile for the main Spanish table olive cultivars (Gordal, Manzanilla, Hojiblanca) cultivated in seven different areas. A total of 15 panelists used a set of descriptors for aroma, taste and mouthfeel [116]. PCA and hierarchical clustering analysis (HCA) were useful to visualize the panel capacity and characterization of samples and their discrimination. The study allowed them to develop a vocabulary for the sensory characteristics of treated green olives from diverse cultivars and production areas. PCA analysis, moreover, permitted them to find correlations among sensory attributes and sample discrimination. A similar paper has been published by the same research group [75] to sensorially describe Manzanilla and Hojiblanca olives processed at the black stage using a list of descriptors able to characterize the product according to varieties, place of growth and duration of shelf life [116]. A total of 14 panelists used a set of descriptors for visual appearance, aroma, flavor, taste and texture. Data were analyzed by MSA. Results indicated the existence of a certain number of attributes that fit the sample discrimination, such as skin sheen, skin red, flesh yellow and others. A relevant effect on the sensory profile was found for the previously cited variables. Sánchez-Rodríguez et al. [76] recently studied the effect of cultivation under regular deficit irrigation (RDI) on sensory quality of fermented Manzanilla table olives. RDI was applied as moderate to severe grade and compared to fully irrigated control trees. Sensory analysis was carried out by 10 trained panelists, who developed an adequate lexicon, or by a consumer acceptance test with 100 consumers [116]. The QDA analysis evidenced an increment of the green olive flavor and a drop of bitter taste in the RDI olives. The customers, who were informed about the irrigation strategy used, preferred the RDI samples, with respect to control, and declared it to be favorable to pay more for these olives. The authors also found that the descriptors driving the consumer acceptance of RDI olives were both gustative, such as bitterness and saltiness, and kinesthetics, such as hardness. The work of Mastralexi et al. [25], also cited in Section 3.1.1., studied the effect of Spanish style processing and a storage period of twelve months on the sensory characteristics of the protected denomination of origin (PDO) “Prasines Elies Chalkidikis” olives prepared at industrial scale. An accredited panel used the attributes related to defects (abnormal fermentation and other defects), taste (acid, bitter and salty) and kinesthetics (crunchiness, fibrousness, and hardness) to evaluate the olives [116]. The sensory panel considered the stored olives as quite satisfactory for texture descriptors and that they could be graded as “extra”.

Research for sensory characterization of treated olives is, thus, at an important level and is to highlight studies directed at developing a vocabulary for descriptors.

#### 3.1.2. Natural Olives

This trade preparation produces olives with a residual bitter taste, and acidic and salty taste and other flavors derived from the microbial fermentation.

Piga et al. [118] evaluated the responses of three Sardinian table olives (Bosana, Manna and Sivigliana sarda) in terms of sensory acceptability after natural fermentation carried out in the Greek-style. Ten untrained laboratory persons performed an informal tasting at 50 days of fermentation and wrote on the presence of off flavors, consistency and crispness, and expressed their preferences. No off flavors were detected by assessors, which found all the cultivars with a balanced taste and satisfactory consistency. The assessors, moreover, considered all the olives excellent and ready to eat after 150 days of brining, preferring the Bosana olives for their best consistency and crispness. The same research group proposed some technological corrections to avoid the main technological problems related to the processing of green natural olives and to improve their sensory properties [119]. The authors controlled and periodically adjusted the following process parameters during the fermentation: brine NaCl concentration, pH, temperature of fermentation and brine level in the fermenters. The same sensory protocol described in [118] was used, and the attribute saltiness was also expressed. The assessors did not detect negative tastes or odors; they judged as excellent the fermented olives after 210 days of brining; and preferred samples obtained with NaCl at 4% for the more intense salty taste. Kanavouras et al. [120] focused their work on the influences of different brines on sensory descriptors of black fermented olives. The authors tested three different brines: a traditional brine with NaCl at 16%; a NaCl-free brine buffered at pH 4.7 with CH_3_COOH (0.05 M) and Ca(OH)_2_ (0.025 M); and a 12.8% NaCl brine buffered at pH 4.3 with CH_3_COOH (0.05 M) and Ca(OH)_2_ (0.025 M). A total of 39 untrained assessors evaluated the fermented samples for appearance and taste, on a 9-point scale, whereas a 3-point scale and a preference was used for the intensity of salt, vinegar, pungency, level of fermentation and unpleasant characteristics. Assessors preferred the olives fermented in the NaCl brine buffered at pH 4.3, because the samples showed a more pungent, fermented and mildly vinegary taste, had a sufficient salty taste and had a low level of unpleasant flavor, while samples processed with a simple NaCl brine were judged as the worst. The authors concluded that consumers seemed to prefer olives with lower NaCl content. An electronic nose was developed by Panagou et al. [77] to sensorially discriminate fermented green table olives on the basis of their aroma compounds. A 15-member panel classified the volatile profiles of the olives as unacceptable, acceptable and marginal, while a specific electronic nose generated a chemical map of the aromatic compounds of fermented samples. All obtained data were analyzed by MSA and artificial neural networks (ANN). The MSA analysis gave a good discrimination between unacceptable, acceptable and marginal samples, while the ANN use resulted in a good performance in discriminating the three classes, as only in two cases of the 66 samples were there misclassifications. The authors suggested that the developed device may be proposed for quality discrimination of green table olives as it had several advantages, such as the low price and the rapidity of analysis. The influences of fruit ripeness (green, turning color and black) and salt concentration (5% and 10% of NaCl) on the sensory properties of Arbequina table olives were evaluated by Hurtado et al. [121]. A 16-member panel judged the olives after fermentation and storage in acidified brine for 45 days according to UNE [122] for color, taste, texture and flesh stone. They also rated any sensory diversity between a sample processed at lab with a 10% NaCl brine and a commercial sample. Results indicated that panelists preferred the olives with a green color and that it was not possible to distinguish commercial samples from laboratory-scale processed olives. Aponte et al. [78] sensorially characterized five naturally fermented table olive cultivars picked at the green stage. Ten judges used a descriptive method [123] with fifteen descriptors for aspect, color, odor and off odor, flavor and off flavor, taste and kinesthetics sensations. Sensory data were affected mainly by cultivar, and the overall assessment was below the imposed threshold of acceptability after 150 days of fermentation. The authors suggested modifying the unit operations to ameliorate olive quality. The use of two NaCl brine concentrations (4% and 7%) was tested in order to see the influence on sensory properties of fermented green olives [79]. Thirty untrained judges expressed their preferences of samples at the end of fermentation by using a paired preference test [124]. The assessors gave positive judgements on both samples but preferred the olives obtained with the lowest NaCl concentration for their lower saltiness and bitterness. Lanza and Amoruso [80] evaluated the sensory characteristics of fermented Itrana table olives, obtained according to two styles differing from the ripening stage at harvest, the green one (Oliva Bianca di Itri) and black one (Oliva di Gaeta). A total of 8–10 panelists used IOOC standards [116] to check for gustatory, kinesthetic and negative sensations of samples. MSA discriminated between samples with or without defects. Assessors graded all samples as “Extra or Fancy,” or as “First, 1st, Choice or Select”. The MSA was able to separate in different areas the defected and un-defected samples and that “Extra or Fancy” olives with a defect higher than 1.0 were judged closer to samples with defects. 

Analysis of literature on sensory properties of natural olives evidenced the lack of studies dealing with the development of a common lexicon that could be proposed for sensorially describing these table olives.

#### 3.1.3. Olives Darkened by Oxidation or Californian-Style

This trade preparation produces olives with no bitter taste, and sensory characteristics of pickled olives if they are fermented before processing.

Lee et al. [81] examined the sensory properties and the preferences of California consumers of sliced black olives produced in several countries from USA (California), Europe (Portugal and Spain) and Africa (Egypt and Morocco). A panel of eight judges selected thirty-four descriptors for aroma, appearance, flavor, taste, texture, and mouthfeel [125], while a consumer test was carried out by 104 consumers that assessed the level of preference of the 20 sliced samples on a nine-point hedonic scale. According to QDA, country of origin well separated samples for aroma and flavor, while appearance and texture were the descriptors that best discriminated the olive products. Californian samples had no flavor defects, while olives produced in other countries revealed gassy, metallic, rancidity and soapy/medicinal defects. The American consumers expressed an important score of acceptability for samples produced in California, probably for their familiarity with the product. The study also revealed that consumers acceptance was driven mainly by the flavor characteristics. García-García et al. [82] evaluated changes in sensory parameters of packed pitted and whole black olives of four Spanish cultivars during a three-year period of storage in simulated marketing conditions. A panel of eight trained people described sensorially the olives using the descriptors of external appearance, odor/flavor and texture on just packed olives and samples stored for 6, 12, 24 and 36 months at ambient temperature [114]. The sensory analysis found significant changes only for surface color of whole olives. The classification of ‘extra’ was attributed to almost all samples. Recently Sanchez et al. [83] sensorially characterized black olives (Manzanilla and Hojiblanca) by comparing the aroma profile with volatile compounds. Fourteen trained panelists assessed eleven odor attributes [81]. Volatiles were extracted with the headspace solid-phase microextraction (HS-SPME) and analyzed by gas chromatography-mass spectrometry (GC-MS). Cultivars were sensorially discriminated only for the briny descriptor. MSA with PLS regression accurately predicted the nutty flavor and permitted the identification of the aroma compounds that highly contributed to the attributes of olives processed at the black stage.

#### 3.1.4. Other Processing Methods and Stabilization Treatments

Gambella et al. [84] studied the influence of different pre-treatments before cabinet drying on sensory properties of green table olives. Olives were subjected to the following pre-treatments: piercing with a steel brush (A), dipping in water at 50 °C for 10 min (B), piercing plus dipping (C), piercing and dipping in a 10% NaCl brine at 50 °C for 10 min (D), untreated as control. Five untrained personnel expressed the intensity of the bitter taste with a three-points scale: 1 = no bitterness, 2 = acceptable bitterness and 3 = unacceptable bitterness. A preference was also given. The sensory test revealed that bitterness was almost absent in D olives and quite strong in olives of the groups A and B. The highest preference was expressed for the least bitter olives (D), that were also judged saltier, with respect to the other groups. In another paper of Piga et al. [85] a preference was expressed on cabinet dried olives belonging to fourteen cultivars. Whole fruits were pre-treated as follows before drying: blanching in 2% NaCl brine at 90 °C for 2 min and room cooling—“blanched olives”; 2 min water blanching at 90 °C plus salting in barrels for 3 days—“salted olives”; skin piercing with a steel brush—“pierced olives”. Ten untrained assessors gave a preference judgement. The sensory analysis revealed that all the olives were appreciated, even if the assessors preferred the salted olives as salt had a masking effect on bitterness. Değirmencioğlu et al. [86] tried to prolong the shelf life of dry-salted Gemlik olives by means of modified atmosphere packaging (MAP) and vacuum sealing. Olives were stored for 7 months at 4 or 20 °C and air packaged olives served as control. A total of 32 untrained assessors evaluated the attributes (color, taste, texture and flesh stone, and overall eating quality) using a nine-hedonic scale [126,127,128]. Assessors rated better MAP and vacuum-packaged olives as they obtained better ratings for rancidity and softness than the control. The sensory profile was not affected by storage temperature, but olives held at 4 °C were rated with the best scores. Pradas et al. [129] proposed the use of HHP (400 MPa and 800 MPa for 5 and 10 min), as an alternative to heat treatment, to sensorially improve “Cornezuelo” dressed olives, a Spanish table olive specialty prepared with the use of some condiments (garlic, fennel, salt and thyme). Sensory analysis was carried out after packaging and at 120, 186, 218, 280 and 335 days of storage by a panel of 6–8 members that are experts in sensory evaluation of table olives, and who scored the olives for odors, flavor defects and overall sensory quality. Only olives treated at 400 MPa for 5 min fulfilled the market requirements after 335 days of storage, as revealed by the sensory analysis. Galán-Soldevilla and Ruiz Perez-Cacho [87] developed a 52 h training method for the PDO Aceituna Aloreña de Málaga quality certification panel. This PDO can be proposed in 3 different styles: cured, that are directly brined for 90 days and then seasoned and packaged; fresh green, that are cracked before brining for 3 days, and after that, seasoned and packaged or stored at low temperature; traditional, that are cracked, brined for 20 days and then consumed or seasoned and packaged [130]. The paper described all the stages involved in the sensory analysis, from recruiting (15 members) to basic and specific training. The panel developed nine specific descriptors for odor (fruity, green, seasoning and lactic), aroma (fruit and seasoning), basic tastes (acid and bitter) and texture (crunchy). The panel also characterized this PDO for its fruity and seasoning odor and aroma, bitter taste and crunchy texture. In a further paper Galán-Soldevilla et al. [89] identified the sensory descriptors that may appropriately distinguish the different styles of this specialty by using nine trained members that selected 15 descriptors for aroma, basic, odor, texture and trigeminal attributes. The evaluated samples were taken by commercial packages. The results showed that the processing style significantly influenced only bitter taste, firmness and odor, while each style resulted in differences for all the descriptors. The PDO fresh green Aceituna Aloreña de Málaga olives were also used to study the effect of addition to brine of zinc chloride (ZnCl_2_ at 0.000%, 0.050%, 0.075% and 0.100%) in increasing their shelf life and improving their sensory properties [90,91]. In a first paper [90] an 18-member panel was used for rating the descriptors of acidity, bitterness, color, crispness, firmness, fibrousness, odor and saltiness [131]. After three months of storage the ZnCl_2_ led, in general, to a better control of microbial spoilage, with respect to the control olives, and olives treated with 0.075 ZnCl_2_ obtained higher scores for acidic taste, color, odor and saltiness. In the second paper [91] the authors confirmed the results of the first one. The work of Malheiro et al. [51], that has been previously cited in Section 3.1.4, investigated the effect of cultivar (Cobrançosa, Madural, Negrinha de Freixo, Santulhana and Verdeal Transmontana) on the sensory characteristics of fermented alcaparras olives. Thirty-tree untrained assessors gave a preference using a nine-point hedonic scale, evaluated aroma, consistency and flavor and rated globally the samples. The consumer test showed a preference for the cultivars Verdeal Transmontana and Negrinha de Freixo, the former for the attributes firm, fleshy and fruity, and the latter for the aroma, while all parameters of the cv. Madural were scored negatively. 

Lanza et al. [93] evaluated the sensory properties of two products derived from processing of Taggiasca olives. Olives were pitted or reduced to a paste, pasteurized inside glass containers filled with extra-virgin olive then stored at room temperature for 18 months. A trained panel used official methods [116] to evaluate the attributes: negative sensations and gustatory sensations. Classification (extra, first, second, not be sold) was done considering the median of defect predominant perceived (DPP). Tasters rated the rancidity defect with a DPP ≤3, which is the threshold for the extra category, for paste olives with up to 18 months of storage, while for pitted olives, this limit was overcome after 12 months. Alves et al. [94] evaluated the possibility of extending the shelf life of cracked green Macanilha olives, which are an appreciated table olive specialty of Southern Portugal, with the addition to packing brines of acids (citric, hydrochloric and lactic) and preservatives acids (sorbic and benzoic). Both categories of chemicals were used one at time, but a combination of lactic and citric acid was also used. An acceptability test was performed after 158 days of packaging by 20 assessors that were regular consumers of table olives. Assessors gave the highest acceptability to olives brined with hydrochloric acid and with the mixture of the other two acids, which was, thus, suggested as an acceptable strategy for the shelf life extension of this table olive specialty. Tokuşoǧlu et al. [39], also cited in Section 3.1.4., investigated the effectiveness of UAD on the sensory characteristics of table olives. Twelve semi-trained panelists evaluated the bitterness of the olives with scores from 1 (unacceptable) to 5 (acceptable) at 7, 14 and 21 days of processing. The UAD operation resulted in olives with a significantly lower bitter taste, with respect to control, thereby highlighting the beneficial effect of the UAD in improving the debittering process. Rodríguez-Gómez et al. [88] used a hot water dipping treatment (5 min at 60 °C) on DPO Aloreña de Málaga olives before brining, to enhance the sensory properties of the fermented olives. Fourteen expert members evaluated the heat treated and non-heat-treated olives for the descriptors acidic, bitterness, crunchiness, hardness and salty [114]. The authors revealed a beneficial effect of the mild heat treatment, as treated olives maintained a better green color, with respect to control, and improved the stability of the samples without imparting them negative sensory attributes. The paper cited in Section 3.1.4 [60] also tested the effect of brine NaCl replacement with CaCl_2_ and/or KCl on sensory characteristics of cracked Maçanilha Algarvia table olives. A sensory panel of fourteen trained judges used the descriptors of acidity, appearance, aroma, bitterness, firmness, flavor and saltiness on a seven-point scale. An overall sensorial evaluation was also carried out. Results of the sensory test evidenced that the olives fermented with 8% NaCl and 4% NaCl + 4% KCl brines obtained the highest scores for flavor and overall attributes, while samples processed with other brine combinations (4% NaCl + 4% CaCl_2_ −4% KCl +4% CaCl_2_ and 2.7% NaCl + 2.7% KCl + 2.7% CaCl_2_) were rated as unacceptable. Another study, on DPO Aloreña de Málaga olives processed with the traditional style, was carried out by Romero-Gil et al. [92], who determined the shelf life of this olive preparation from a sensory point of view. Olives were packaged in appropriate containers filled with a brine containing acids and preservatives and sensorially checked at 0, 6, 20, 42, 74, and 131 days. A consumer panel consisting of 35 members used specific descriptors related to Aloreña de Málaga fruits and to IOOC method [116] and expressed a global evaluation of the olive quality and acceptability by using yes (olives good for purchasing) or not (poor quality). Data were analyzed by MSA and showed the highest acceptance for olives with a shelf life from 6 to 42 days, while a drastic decrease in sensorial quality was found at 131 days, as the willingness-to-buy attribute was reduced to 50%. Rodrigues et al. [95] developed an electronic tongue for monitoring the debittering of previously described alcaparras olives. Data obtained by the electronic tongue were correlated with the sensory descriptors of bitter, pungent, salty and sweet recorded by 8 trained judges following the IOOC official regulations [116]. Data obtained were analyzed with multivariate statistical techniques and evidenced that the electronic tongue is effective at evaluating changes in bitter, pungent and sweet intensities and may be proposed as a tool with different useful characteristics, such as rapidity of analysis and low environmental impact. The authors also stated the possibility to use the electronic tongue for tasting purposes.

#### 3.1.5. Comparison among Different Trade Preparations and Styles

Only four papers were found dealing with this topic.

Panagou et al. [132] studied the sensorial characteristics of retail table olives. Sixty-nine different samples processed as green treated olives, black natural olives and other samples were considered. A ten-member panel evaluated the olives for the specific attributes of acidic taste, bitterness, crispness, odor, saltiness and overall eating quality on a 1–10 scale [127]. Assessors evidenced that the green olives were not different, and that they were principally characterized by a sufficient acidity, an adequate bitterness and a satisfactory crispness and odor; more variability was found for black olives that showed more remaining bitterness, with respect to green ones adequate acidity and odor, and high NaCl content. A higher residual bitter taste was found on dry-salted olives, with respect to other samples and were perceived as too much salty. The previously cited paper of Valenčič et al. [41] studied the sensory profile at different processing times (60 and 180 days) of Slovenian table olives prepared according to a traditional regional and modified Spanish style. Nine trained assessors evaluated the sensory characteristics of olives [113] and used the descriptors for saltiness, bitterness, sourness, hardness and fibrousness. The authors found that the intensity of bitterness, fibrousness, hardness and sourness of both cultivars were higher in the traditional technology, with respect to Spanish style samples, which were judged not suitable to be classified as Slovenian table olives. Lanza et al. [64] evaluated the sensory properties of Itrana table olives fermented for 8 and 12 months at the green (Oliva bianca di Itri) or black stage (Oliva di Gaeta), according to the place of harvest, the maturity stage and the preparations styles reported in Section 3.1.5 by the same paper. The IOC method [116] was used for sensory analysis with the evaluation of negative, gustatory and kinesthetic sensations. The DPP was also used as reported by Lanza et al. [93]. Assessors did not find defects for green and black olives processed with the double-salting methods that were, thus, rated as the best. Lanza and Amoruso [133] recently monitored on a regular basis the capacity of every assessor and of the entire panel that applied criteria and procedure of the official IOOC method for table olives [116]. Olives were sensorially evaluated by 8 expert assessors. Univariate and MSA of data were applied. Results indicated that the panel well agrees for hardness, while a case-to-case analysis was needed for other attributes.

### 3.2. Influence of Starters

The fermentation process of table olives is aimed at stabilizing the product by reducing the pH and changing positively the olive sensory properties. Brine fermentation with indigenous microbiota, although it is widely used, could be responsible for spoilage and pathogen microorganism growth in the first phases of fermentation. The use of starter cultures made up of LAB, Y or their mix may help in preventing the cited problems and producing high quality products. For these reason, extensive research has been carried out in the last 20 years, mostly for natural olives, which need the development of specific LAB starters that can grow in the presence of specific polyphenol inhibitors derived from the fruit flesh. We will review the literature starting from papers dealing with natural olives.

#### 3.2.1. Natural Olives

Marsilio et al. [96] evaluated the sensory quality of Ascolana tenera olives that were subjected to different pre-harvest irrigation regimes and that were fermented with a LAB. The irrigation regime consisted of a rainfed control, two regimes with water depth of 33% and 66% of the estimated crop evapotranspiration (ETc) from the seed hardening stage and a four one with 66% of ETc during the whole season. Olives were fermented with a LAB made with *Lactobacillus plantarum* strain or with indigenous microbiota. An eight-member panel made a QDA analysis and assessed the olives after seven months of fermentation for color, odor, acid/sour, bitter, firmness and crispness [112]. The control olives showed overly high bitter taste, firmness and sourness, and thereby were judged as not marketable. The LAB samples were more appreciated than non-inoculated ones, as they were found to have less bitter taste, a higher odor intensity and good textural attributes. In another study olives with a pre-harvest treatment with copper-based products and kaolin were processed using two selected LAB strains (*L. casei* T19 and *L. plantarum* UT2.1) and sensorially evaluated at the end of fermentation [97]. Un-treated olives and olives fermented with indigenous population were used as control. Eight trained panelists used an official method to assess the descriptors of taste and texture on a 1–10 scale [113]. Panelists judged the treated olives and the not treated ones fermented by *L. plantarum* the best for acidic and salty tastes and for crunchiness. Additionally, there was not always a correspondence between sensory and chemical data. Randazzo et al. [98] tested LAB and probiotic LAB strains on sensory characteristics of green olives. The olives were fermented as follows: spontaneous fermentation (control), inoculation with probiotic *L. rhamnosus* H25, inoculation with commercial probiotic *L. rhamnosus* GG, inoculation with *L. plantarum* GC3 and *L. paracasei* BS21 and inoculation with *L. plantarum* GC3 plus *L. paracasei* BS21 plus *L. rhamnosus* H25. A panel of eleven trained judges used 15 descriptors to evaluate the ready-to-eat olives. Results evidenced that sensory characteristics were cultivar dependent. De Angelis et al. [108] used an omics procedure to study the capacity of LAB and Y starters to enhance the sensory properties of black Bella di Cerignola table olives. The authors used four combinations, a commercial *L. plantarum* strain (S), the same S plus the autochthonous Y *Wickerhamomyces anomalus* DiSSPA73 (SY), the autochthonous *L. plantarum* DiSSPA1A7 and *Lactobacillus pentosus* DiSSPA7 (SYL), while the fourth fermentation was carried out with the indigenous microbiota and served as control. A panel of eight trained judges used seven descriptors to evaluate the olives (crunchiness, bitter, acid, sweet, salty, flavor and off flavor) [116]. The panelists ranked better the olives fermented with starters, especially the SYL, with respect to the control that obtained the lowest values for crunchiness and olive flavor and the best evaluations for the descriptors acid, bitter and off flavor. All the started olives were judged ready for consumption after 90 days of fermentation. Campus et al. [99] compared the effect of fermentation for 156 days driven by a single strain of *L. plantarum* (SSL) and by a mix of *L. pentosus* strains (SIE), which were isolated from previous successful fermentations, with a control carried out with indigenous microbiota (NF). Sensory analysis of the fermented green olives was performed by eight trained assessors [134] that were calibrated for the “bitterness” descriptor with a standard of reference and commercial olives and olive pastes [116]. The assessors found that the two samples obtained with LAB were debittered at the end of processing, while control olives needed 12 months, thus, according to the authors, the use of these starters may be suggested to reduce fermentation times and production costs, and to limit spoilage risk, improve the process control and standardize the product. The same group of authors integrated the above-cited work with a study aimed at focusing on dynamics of microbial growth and at developing a wider set of sensory descriptors [100]. Seven trained assessors sensorially evaluated the olives using the descriptors of acidity, bitterness, crunchiness, fibrousness, freestone, hardness and saltiness [116]. The sensory analysis on the ready-to-eat olives showed that the SIE starter resulted in olives with a sensory profile very close to natural-style samples, with respect to SSL. Martorana et al. [101] proposed an innovative approach based on the wine-technology of “pied de cuve” to sensorially enhance green table olives. The preparation of pied de cuve involved a table olive fermentation of 10 days with both indigenous microbiota (control) or the autochthonous LAB strain *L. pentosus* OM13. These pre-fermented brines were used to carry out the experimental fermentations and compared with the two controls, one made with spontaneous fermentation, the other with use *L. pentosus* OM13. Sensory analysis was performed after 200 days by 12 trained assessors that used nine descriptors for odor (green olive aroma), rheological characteristics (crunchiness), taste (sweet, acid, bitter, salty and complexity), off odor and off flavor [123]. Data analyzed by MSA revealed that the use of spontaneously fermented pied de cuve resulted in olives with the highest scores of sensory complexities and with the absence of any off odors and off flavors. The effects of mechanical harvesting on sensory quality of olives has been investigated for the first time by Martorana et al. [102]. The autochthonous LAB *strain L. pentosus* OM13 was used for fermentation, while uninoculated olives were used as control. Manually harvested drupes fermented either with the LAB strain and with an indigenous microbiota served as controls. Twelve judges used 15 descriptors for the analysis of external aspect, odor, taste and off flavors [123]. MSA of data evidenced that the mechanically harvested and LAB fermented olives were sensorially similar to the manually harvested olives, thereby suggesting that mechanical harvesting and fermentation with LAB starter could substitute the manual harvesting for table olive processing. Campus et al. [103] developed an automated pilot plant (CF), in which a LAB starter was used, and compared the sensory attributes of the obtained fermented olives with samples processed with spontaneous fermentation (NF). The pilot plant was equipped with a control of: temperature, brine, internal pressure of reactor, flow rate of the circulation pump, pH, dissolved CO_2_ and brine concentration. Eight trained assessors performed the QDA [116,134] with thirteen descriptors. Assessors evaluated the bitterness until it was reached a determined commercial bitter level, based on a retail sample. CF samples obtained the same level of bitterness of commercial sample after only 90 days, while NF olives had a significantly higher bitter taste than commercial sample after 180 days. Authors are encouraged to suggest this approach as the reduction of fermentation times may be considered as environmentally friendly. Pino et al. [104] evaluated the differences in sensory properties of green table olives fermented in brines with different NaCl contents (4%, 5%, 6% and 8%) and fermented with LAB starters (*L. plantarum* UT2.1 and *L. paracasei* N24) or indigenous microbiota. A ten-trained panel rated olives for negative sensations and used the descriptors for gustatory and kinesthetic sensations [116]. Panelists gave also an overall acceptability score. Panelists rated the LAB fermented olives with a significantly high overall acceptability score and the sample brined with the 5% NaCl obtained the best appreciation. The authors conclude that the formulation of LAB fermented olives with reduced NaCl is healthier and could be suggested to avoid risks in people suffering from hypertension. Randazzo et al. [105] used six LAB starters constituted by L. *plantarum*, *L. paracasei* and *L. pentosus*, alone or in combination, to process for 180 days Nocellara Etnea olives and investigated their effects on the olive sensory quality. A non-inoculated control was used as reference. A trained panel of 12 judges used 15 descriptors to evaluate the olives [73]. Results indicated significant differences in bitterness, bright, crunchiness, green color, green olive aroma and juiciness for LAB samples, while the control olives had the highest bitterness value. The authors found that fermentation with a combination of *L. plantarum* + *L. paracasei* resulted in olives with the lowest bitter taste. The LAB processed olives, moreover, obtained the best value of overall quality. Romeo et al. [106] studied the differences in the sensory properties of fermented Aitana and Caiazzana black olives and Nocellara del Belice turning color olives. Olives were processed with a commercial LAB (*L. plantarum* Lyoflora V3, Sacco) or with indigenous microbiota. A panel of 12 trained tasters used eight descriptors to evaluate the fermented olives [116]. Panelists found that all the tested cultivars had good sensory characteristics, and gave the highest scores for flesh consistency and crunchiness to Nocellara del Belice olives. Sensory differences among cultivars were mainly explained by the descriptors of acid, bitter and hardness. Pino et al. [107] used a sequential inoculum procedure with LAB starters with the aim to evaluate the sensory characteristics of green Nocellara etnea olives brined at 5% and 8% NaCl for 120 days. The inoculation procedure consisted of using at the beginning the α-glucosidase positive strain *L. plantarum* F3.3 and adding after 60 days the potential probiotic *L. paracasei* N24 strain. A control test with no starter was also considered. Ten trained panelists described and rated the olives for attributes linked to gustatory, kinesthetic and negative sensations, and also assigned an overall quality score [116]. The panelists did not perceive negative sensation and did not detect significative differences for crunchiness, fibrousness or hardness among samples. The control olives obtained the highest scores for acidity, while the highest bitter taste was scored in the samples obtained without *L. plantarum* addition. The samples at 5% and 8% NaCl added with the *L. plantarum* strain received the highest overall acceptability. Another research group proposed the sequential inoculation approach by using LAB and Y starters, as an alternative to natural fermentation, to improve the sensory properties of Conservolea and Kalamata olives [109]. The experimental plan considered the use of LAB followed by yeast (LY), the opposite (YL), the use of Y and LAB (MIX) and an indigenous fermentation (Sp). The LAB were *Leuconostoc mesenteroides* K T5-1 and *L. plantarum* A 135–5; the Y were *S. cerevisiae* and *Debaryomyces hansenii*. Fifty-one untrained and seven trained assessors carried out acceptability and descriptive tests on olives after 105 days of fermentation. Panelists scored olives for acidity, bitterness, hardness, odor and saltiness, and gave an overall score. Kalamàta olives obtained the best scores for aroma and overall acceptability when the Y+LAB and MIX inoculations were used, while Conservolea olives showed the same results when LY were inoculated. The use of only Y starters has been recently proposed by Ciafardini and Zullo [110] with the aim to study their effects on the sensory properties of black Taggiasca olives fermented with acidified brined with 8 and 12% NaCl solutions. The Y species *Candida adriatica* 1985, *C. diddensiae* 2011, *Cyteromyces matritensis* 2005, *Nakazawaea molendini-olei* 2004, *S. cerevisiae* 2046 and *Wickerhamomyces anomalus* 1960 fermented the brines for 120 days along with an inoculated control. A panel of eight judges used a QDA to describe olives for gustatory and kinesthetic attributes. The panelists detected significant differences for bitterness, saltiness and hardness among samples [116]. The bitter taste was significantly lower in olives fermented with *C. diddensiae* 2011, *C. adriatica* 1985, and *W. anomalus* 1960. The salt flavor was higher in 12% NaCl processed olives, while no defects were detected in the samples. The authors suggest that the best combination in terms of sensory quality, may be obtained with the use of Y on acidified brines at the highest NaCl concentration.

#### 3.2.2. Treated Green Olives or Spanish Style

Aponte et al. [111] successfully attempted to obtain a more predictable fermentation by using autochthonous LAB cultures. Olives were harvested from irrigated and not irrigated fields and a preliminary LAB isolation led to the isolation of 88 different strains. The authors used *L. pentosus* OM13, alone or in combination with a *L. coryniformis* strain, to enhance the quality of olives, while a non-inoculated fermentation was used as control. Sensory analysis was done after 60 and 120 days of brining by a panel of 10 trained judges who used fifteen descriptors for aspect, flavor, odor, tactile in mouth, texture and overall judgement [123]. A nine-point scale was used for ratings. Data highlighted that *L. pentosus* improved the sensory characteristics of olives, with respect to control samples. In the work already cited in Section 3.2 by Tataridou et al. [45], it was verified the efficacy of the autochthonous oleuropeinolytic strains of *L. plantarum* on sensory properties of the fermented olives, and compared to fermentation with spontaneous microbiota. A nine-member panel evaluated the olives for the descriptors color, odor, flavor (acid, bitter, salty), firmness and crispness. A global assessment score was also expressed. The panelists did not find any statistical difference between control and LAB fermented olives for bitterness and saltiness. The same LAB *L. pentosus* OM13 was used by Martorana et al. [135] both to enhance the fermentation of treated green olives and to study its effect on sensory quality of processed olives, in comparison with two controls (one fermented by spontaneous microbiota and another one with the addition of the studied strain). To improve the growth potential of the LAB culture the following procedures were applied: addition of lactic acid to bring brine pH at 7.0 (IOP1); lactic acid and a nutrient adjuvant (IOP2); the same as IOP2, but brine acclimatization for 12 of the LAB strain before inoculation (IOP3). Twelve judges carried out the sensory analysis after 195 days of processing. A descriptive method [123], including 16 descriptors, was used. A MSA analysis of data revealed that the IOP2 and IOP3 were very close regarding the positive characteristics of complexity (odor and taste), green olive aroma and overall acceptability, while the control olives showed the negative descriptors of bitter, astringent taste and off odors. A *L. pentosus* strain (LP99) isolated in brine of Manzanilla was also recently tested by de Castro et al. [136] and compared with a spontaneous fermentation control. In this case the authors sensorially analyzed the brines to check if differences in concentration of 4-ethyl phenol, which causes off odors, between LAB fermented and control olives resulted in perceivable differences in odor. To that end, 18 trained panelists performed a triangle test. The authors found that, despite the higher concentration of 4-ethyl phenol in inoculated olives, with respect to the control, panelists did not find sensory differences between the two theses, probably because its concentration was below the odor threshold. 

#### 3.2.3. Comparison among Different Trade Preparations and Styles

The work of Marsilio et al. [137] investigated the effect of the LAB strain *L. plantarum* (LAB B1–2001) on the sensory characteristics of Greek style-olives (GSP-i) in comparison to a control fermented with indigenous microbiota (GSP-s) and olives processed with the Spanish-style (SSP). A trained panel of 17 members evaluated the fermented samples for the descriptors of odor, bitterness, firmness and crispness [43]. Sensory analysis showed that SSP olives were less bitter, crisp and firm than both GSP samples. GSP-s obtained the higher bitter and lower odor scores, with respect to GSP-i. MSA of data by PCA well discriminated GSP-s from GSP-i olives. 

## 4. Conclusions

The increasing consumer demand for foods with high contents of phytochemicals has stimulated the industry and research to develop new products that meet this requirement, or to study more deeply the existing ones. Table olives fall surely into the second category, and are one of the basic foods in the human diet, especially in Mediterranean countries. Their balanced fatty acids and the presence of important amounts of polyphenols and fibers and the contemporary sensory peculiarities of the very high number of preparations may further improve their use in the future. For these reasons, during the last two decades, researchers have been focusing their studies on the effects of pre-harvest, cultivar and processing factors on the nutritional and sensory properties of table olives. The review has pointed out the preeminent role of trade preparations and processing styles mainly on polyphenols and lipids. Fermentation with the natural style has been confirmed as the best preparation for maintaining the highest content of polyphenols and tryacilglicerols, while no comparative study comparing the effects of the main trade preparations on fat compounds has been reported. Despite the number of papers discussing this topic, there is a real need to focus future studies on the *in vivo* effects of these supposed nutritional claims; thus, it would be advisable to carry out multidisciplinary studies that compare technological aspects with health benefits. Moreover, more effort should be made to study new debittering technologies that are able at the same time to reduce the process time and maintain the nutritional and sensory quality of olives while assuring more sustainability from an economical and environmental point of view.

The review has also revealed the increasing interest over the last two decades of researchers in describing table olives sensorially. This topic has been deeply studied by using internationally recognized sensorial procedures, and the data obtained, treated with rigorous statistical approaches, gave important knowledge for the discrimination and quality evaluations of the different trade preparations. A further goal of sensory analysis could be that of developing a unique, world-wide accepted test for each trade preparation, as in the case of virgin olive oils. 

Finally, the importance of using starters to reduce processing times and improve the overall quality of olives has been thoroughly reviewed, and the need to find suitable commercial cultures in the future has emerged.

## Figures and Tables

**Table 1:** Effect of trade preparation and processing style on nutritional quality of table olives.

Compound Class	Trade Preparation/Processing Style/Starters (LAB*, Y)	Olive Cultivar	Olive Ripening Stage	Nutritional Results Related to the Compound Class (Results Related to Other Compounds)	References
Polyphenols	Treated	Manzanilla	Green	A moderate level of irrigation increased oleuropein and oleoside diglucoside in table olives	Sánchez-Rodríguez et al. [24]
		Prasines Elies Chalkidikis	Green	Hydroxytyrosol, tyrosol and oleoside-11-methyl ester at higher concentrations in treated olives with respect to fresh ones (decrease of squalene in brine stored olives)	Mastralexi et al. [25]
	Natural	Various	Black	Greek-style olives are a good source of polyphenols	Boskou et al. [28]
		Bella di Cerignola	Black	Presence of hydroxytyrosol acetate, caffeoyl-6′-secologanoside and comselogoside with good bioaccessibility	D’Antuono et al. [31]
		Cornezuelo	Green	Prevalence of oleuropeine and comselogoside isomers, polyphenols retain a good antioxidant activity after digestion (high Ca content)	Fernández-Poyatos et al. [34]
		Eight different Greek cultivars	Black	High concentration of the nutritional important compound 3,4-dihydroxyphenylglycol	Rodríguez et al. [35]
	Dried	Thassos	Black	Storage at 20 °c of dried olives allowed the best retention of polyphenols	Mantzouridou et al. [33]
		Majatica di Ferrandina	Black	High content of biophenols	Lanza et al. [37]
	Other (Water debittering)	Megaritiki	Black	New compounds were detected, two are unique for the species (rengyoxide and cleroindicin C) and one for table olives (haleridone)	Mousori et al. [38]
	Other (High Pressure Processing)	Different Turkish cultivars	Black	Increase of phenolics following the HPP treatment	Tokuşoğlu et al. [39]
	Mixed (Natural and drying with salt)	Meski, Chemlali, Besbessi, Tounsi	Black	Olives of Tunisian market have an important content of polyphenols	Ben Othman et al. [40]
	Mixed (Water dipping and Spanish Style)	Istrska belica, Storta	Green	Debittering with water dipping resulted in higher biophenol content in olives	Valenčič et al. [41]
	Mixed (Five different processing styles)	Nine different Greek cultivars	Black	The highest oleuropein content was found on dry-salted Throuba Thassos olives	Zoidou et al. [42]
	Mixed (Darkened by oxidation and drying with salt)	Manzanilla, Mission, Throuba Thassos	Black	Confirms results of Zoidou et al. [42]	Melliou et al. [43]
	LAB (Natural)	Frantoio, Carolea, Coratina, Leccino	Black	LAB produced tissue skin degradation with consequent higher polyphenol leakage and reduced debittering time	Servili et al. [44]
		Kalamata, Chalkidikis	Black, green	LAB fermented olives had a significantly higher content in phenols, especially hydroxytyrosol and tyrosol	Tataridou et al. [45]
	LAB + Y (Natural)	Taggiasca	Black	Polyphenol loss from flesh to brines depend on process temperature and not on starter use	Pistarino et al. [46]
	Y + LAB (Natural)	Cellina di Nardò, Conservolea, Kalamàta, Leccino	Black	Olives are rich in polyphenolic compounds (rich in MUFA, polyphenols, tocopherols and triterpenic acids)	Durante et al. [47]
		Bella di Cerignola, Termite di Bitetto, Cellina di Nardò	Black	The use of starters allowed to obtain olives with high content of tyrosol and hydroxytyrosol that were up to eight times higher with respect to the virgin olive oils obtained by the same olives	D’Antuono et al. [48]
	Y (Natural)	Picual, Manzanilla, Kalamàta	Black, green	The Y allowed an increase in hydroxytyrosol, tyrosol and verbascoside on the olives, with respect to the control sample, thus improving the nutritional value	Tufariello et al. [49]
Fatty acids	Treated	Meski, Picholine, Sayali	Black, cherry, green	Decrease of FA and increase of the PUFA/SFA ratio after processing (α-tocopherol decreased after fermentation, mainly in black olives)	Sakouhi et al. [19]
		Intosso d’Abruzzo	Green	Optimal MUFA/SFA ratio (appreciable amounts of polyphenols, α-tocopherol, minerals and fibre)	Lanza et al. [21]
		Manzanilla, Hojiblanca		PUFA/SFA ratio lower than 0.5	Lopez-Lopez et al. [22]
		Manzanilla	Green	High PUFA content in olives grown under moderate irrigation regime	Cano-Lamadrid et al. [23]
	Natural	Maçanilha, Cobrançosa, Galega	Black	A serving size of Maçanilha olives provide 13.1% of the recommended daily intake of PUFA (important content of dietary fiber and polyphenols)	Pires-Cabral et al. [29]
		Cellina di Nardò, Conservolea, Kalamàta, Leccino	Black	High content of MUFA	Durante et al. [47]
	Oven-dried	Ferrandina	Black	PUFA/SFA ratio higher than 0.5 (dried fruits contained appreciable amounts of phenols and tocopherols)	Lanza et al. [37]
	Other (Alcaparras)	Not reported	Green	Oleic acid content up to 81% (lower content of Vitamin E if compared to olives prepared with other styles)	Sousa et al. [50]
		Cobrançosa, Madural, Negrinha de Freixo, Santulhana, Verdeal, Transmontana	Green	Content of some fatty acids and of SFA, MUFA and PUFA permitted a statistical discrimination among cultivars	Malheiro et al. [51]
	Mixed (Treated, natural, darkened by oxidation)	Alorena, Arbequiña, Cacereña, Carrasqueña, Gordal, Hojiblanca, Manzanilla, Verdial	Green, black	The fat profile was useful to discriminate olive cultivars	López-López et al. [52]
Minerals	Cracked	Aloreña	Green	Packing brines with combinations of CaCl_2,_ KCl and NaCl resulted in significant reduction of flesh Na content, with respect to the traditional packed product	Moreno-Baquero et al. [53]
		Maçanilha Algarvia	Green	Brines at 4% NaCl + 4% KCl gave olives with increased K and reduced Na contents (lower fat, similar dietary fiber, phenolic compounds and Ca content with respect to the control brine (8% NaCl)	Saúde et al. [54]
	Mixed (Treated, water dip, scratched plus CaCl_2_ dipping and reduced salt brines)	Domat	Green	Reduced salt content in scratched olives processed in low-salt brines	Savas et al. [55]
Triterpenic acids	Mixed (Treated, natural, darkened by oxidation)	Seventeen different cultivars	Black, green	Natural-style olives have the highest content of triterpenic acids, with respect to the other trade preparations and to virgin olive oil	Romero et al. [56]
Fibres	Mixed (Natural, darkened by oxidation, dried)	Douro, Hojiblanca, Cassanese, Conservolia, Taggiasca, Thasos	Black, green	High content of fibers in all samples	Jiménez et al. [57]

*LAB= lactic acid bacteria; Y=yeast; FA= fatty acids; MFA=monounsaturated fatty acids; PF=polyunsaturated fatty acids; SFA=saturated fatty acids.

**Table 2:** Effect of trade preparation and processing style on sensory quality of table olives.

Trade Preparation/Processing Style/Starters (LAB*, Y)	Olive Cultivar	Test Used	Descriptors	Main Results	References
Treated	Not reported	QDA	Acidity, bitterness, color, saltiness, intensity and persistency of nasal aroma	Color, firmness, acidity and saltiness best characterized the olive	González et al. [68]
	Nocellara messinese	QDA	Appearance, color, odor, flavor, texture, overall	Olives treated with CO_2_ are more acidic that control	Marsilio et al. [69]
	Çelebi, Domat, Kaba, Ayvalık	QDA Preference	Appearance, aroma, flavor, texture	Cultivars were sensorially different	Yilmaz et al. [70]
	Gordal	QDA	Abnormal fermentation type, cooking effect, earthy, metallic, musty, rancid, soapy, winey-vinegary; acidity, bitterness, saltiness; crunchiness, fibrousnesses, hardness	Saltiness was significantly related to NaCl and KCl levels; bitterness, hardness, fibrousness, and crunchiness were related to the CaCl_2_ percentage	Moreno-Baquero et al. [71]
	Manzanilla	QDA	As previous	Decrease in saltiness and increase in bitterness at increasing Ca amounts in the pulp. Ca content highly correlated with some kinaesthetic and taste attributes	López-López et al. [72]
	Manzanilla	QDA Acceptability	Color and size; aftertaste, bitter, green olive flavor, salt, sour, sweet; crunchiness, fibrousness, hardness, pit removal	Olives grown under soft stress conditions were preferred and rated as the best for the more important descriptors	Cano-Lamadrid et al. [73]
	Gordal, Manzanilla, Hojiblanca	QDA	Acetic acid, grass, green fruit, hay, lactic acid, lupin, ripe fruit, musty, winery; alcohol, bitter, salty, sour; astringent, piquant, pungent	Development of a lexicon for the sensory characteristics of Spanish-style olives	López-López et al. [74]
	Manzanilla and Hojiblanca	QDA	A total of 33 descriptors (see paper)	A certain number of the descriptors attributes fit sample discrimination	López-López et al. [75]
	Manzanilla	QDA Acceptability	As in López-López et al. [72]	Increase of the green olive flavor and decrease of bitter taste in olives subjected to deficit of irrigation. Consumer preference for the same samples.	Rodríguez et al. [76]
Natural	Not reported	QDA	Abnormal fermentation, cooking effect, musty, rancid	Data analysis gave a good discrimination between unacceptable, acceptable and marginal samples and evidenced that olives could be discriminated by an electronic nose developed in the study	Panagou et al. [77]
	Brandofino, Castriciana, Manzanilla, Nocellara del Belice, Passalunara		Brightness, intensity of the green color; odor of green olives, off odor; crispness, easy peeling, juiciness; acid, bitter, salt, sweet; astringent; green olive flavor, off flavor; overall	Sensory data were affected mainly by cultivar and the overall assessment was below the imposed threshold of acceptability after 150 days of fermentation	Aponte et al. [78]
	Tonda di Cagliari	Preference		Assessors preferred olives obtained with the lowest salt concentration for the lower salt and bitter taste	Fadda et al. [79]
	Itrana	QDA	Butyric fermentation, putrid fermentation; acid, bitter, salty; crunchiness, fibrousness, hardness	All sample were rated as “Extra or Fancy”, or as “First, 1st, Choice or Select”. The analysis was able to separate in different areas the defected and un-defected samples	Lanza and Amoruso [80]
Darkened by oxidation	Not reported	QDA Degree of liking	A total of 34 descriptors (see paper) of appearance, aroma, flavor, taste and texture	The QDA showed that country of origin well separated samples for showed that aroma and flavor, while appearance and texture were the descriptors that best discriminated the olive products. The American consumers expressed an important score of acceptability for samples produced in California	Lee et al. [81]
	Cacereña, Gordal, Hojiblanca, Manzanilla	QDA	Brightness, skin defects, surface color; acid, bitter, salty; abnormal fermentation, other defects; crunchiness, fibrousnesses, hardness, pit release, skin strength; metallic taste, soap taste, typical flavur	The sensory analysis found significant changes only for surface color of whole olives. The classification of ‘extra’ was attributed to almost all samples	García-García et al. [82]
	Hojiblanca, Manzanilla	QDA	Alcohol, artificial fruity/floral, briny, cheesy, earthy/soil-like, fishy/ocean-like, natural fruity/floral, nutty, oak barrel, sautéed mushroom, vinegary	Cultivars were sensorially discriminated only for the briny descriptor. Analysis of data accurately predicted the nutty flavor and permitted the identification of the aroma compounds volatiles that highly contributed to the attributes of olives processed at the black stage	Sanchez et al. [83]
Dried (hot air or salt)	Ascolana Tenera	Preference		The highest preference was expressed for the least bitter olives, that were also judged saltier, with respect to the other samples	Gambella et al. [84]
	Various (see paper)	Preference		Assessors preferred the salted olives as salt had a masking effect on bitterness	Piga et al. [85]
	Gemlik	QDA	Black, black-brown, brown; bitterness, off flavor, rancidity, saltiness; softness, pit-flesh detachment; overall eating quality	MAP and vacuum-packaged olives as well as those stored at 4 °C obtained the best scores	Değirmencioğlu et al. [86]
Other (Cured, fresh green, traditional)	Aloreña de Málaga	QDA	Descriptors were developed in the work	The panel developed nine specific descriptors: odour (fruity, green, seasoning, lactic), aroma (fruit, seasoning), basic tastes (acid, bitter), texture (crunchy)	Galán-Soldevilla and Ruiz Perez-Cacho [87]
	Aloreña de Málaga	QDA	Acidic, bitterness, crunchiness; hardness, salty; appreciation of defects, darkening, overall acceptability	Olives subjected to a hot water dipping maintained a better green color, with respect to the control	Rodríguez-Gómez et al. [88]
Other (Fresh)	Aloreña de Málaga	QDA	Descriptors were developed in the work	Assessors selected 15 descriptors for aroma, basic, odor, aroma, trigeminal and texture attributes. The processing style significantly influenced fruit odor, bitter taste, firmness and odor, while each style resulted in differences for all the descriptors	Galán-Soldevilla et al. [89]
	Aloreña de Málaga	QDA	Acidity, bitterness, saltiness; color; crispness, firmness, fibrousness; odor	Olives treated with 0.075 ZnCl_2_ obtained higher scores for acidic taste, color, odor, saltiness	Bautista-Gallego et al. [90]; Bautista-Gallego et al. [91]
Other (Traditional)	Aloreña de Málaga	QDA Acceptability	Acidic, bitter, salty; crunchiness, hardness, appreciation of external damages and any kind of defects, browning	The highest acceptance was obtained by olives with a shelf life from 6 to 42 days, while a drastic decrease in sensorial quality was found at 131 days	Romero-Gil et al. [92]
Other (Pitted, reduction to a paste)	Taggiasca	QDA	Abnormal fermentation, cooking effects, musty, rancid, other defects present; acid, bitter, salty	Assessors rated the rancidity defect with a defect predominant perceived <3, which is the threshold for the extra category, for paste olives up to 18 months storage, while for pitted olives this limit was overcome after 12 months	Lanza et al. [93]
Other (Cracked)	Maçanilha	Acceptability		Assessors gave the highest acceptability to olives brined with HCl and with the mixture of citric and lactic acid	Alves et al. [94]
Other (Alcaparras)	Cobrançosa, Negrinha de Freixo		Bitter, pungent, salty, sweet	Data of sensory analysis were corelated with those obtained with an electronic tongue and revealed that this device is effective in monitoring the changes in bitter, pungent and sweet intensities	Rodrigues et al. [95]
LAB (Natural)	Ascolana Tenera	QDA	Acid/sour, bitter; color; odor; crispness, firmness	The LAB olives were more appreciated than non-inoculated ones, for their less bitter taste, a higher odor intensity, and good textural attributes	Marsilio et al. [96]
	Nocellara Etnea	QDA	Acid, bitter, salty, sweet; crunchiness, fibrousness, hardness	Panelists judged olives treated and fermented by LAB the best for acidic and salty tastes and for gave the highest scores for acidity, crunchiness and saltiness	Randazzo et al. [97]
	Giarraffa, Grossa di Spagna	QDA	Bright, green color; green olive aroma, off odor; crisp, easy stone, juicy; acid, bitter, salt, sweet; astringent; green olive flavor, off flavor; overall	Results evidenced that the sensory characteristics were cultivar dependent	Randazzo et al. [98]
	Tonda di Cagliari	QDA	Bitterness	Samples obtained with LAB were debittered at the end of processing, while control olives needed 12 months	Campus et al. [99]
	Tonda di Cagliari	QDA	Acidity, bitterness, saltiness; crunchiness, fibrousness, freestone, hardness	The use of *L. pentosus* resulted in olives with a sensory profile very close to the natural-style samples, naturally fermented ones, with respect to *L. plantarum*	Communian et al. [100]
	Nocellara del Belice	QDA	Green olive aroma; crunchiness; acid, bitter, complexity, salty, sweet; off odor, off flavor	The use of pied de cuve resulted in olives with the highest scores of sensory complexities and with the absence of off-odors and off flavors	Martorana et al. [101]
	Nocellara del Belice	QDA	Green color intensity; green olive aroma, off odors; crispness, easy stone detachment; astringent, bitter, complexity, juicy, salt, sour, sweet; off flavors	Mechanically harvested and LAB fermented olives were sensorially like the manually harvested olives	Martorana et al. [102]
	Tonda di Cagliari	QDA	Acetic, acid, bitter, fruity, mushroom, saltiness, silage; astringent, crunchiness, fibrousness, fleshy, freestone, hardness, juiciness	Samples fermented in an automated pilot plant obtained the same bitterness of commercial sample after 90 days, while control olives had a significantly higher bitter taste after 180 days	Campus et al. [103]
	Nocellara Etnea	QDA	Cooking effect, earthy, metallic, musty, rancid, soapy, winey-vinegary; acidity, bitterness, saltiness; crunchiness, fibrousness, hardness	LAB fermented olives obtained the significantly highest overall acceptability score and the sample brined with the 5% NaCl obtained the best appreciation	Pino et al. [104]
	Nocellara Etnea	QDA	Green color, bright; green olive aroma, off odor; green olive flavor, off flavor; acid, bitter, salty, sweet; crunchiness, easy stone separation, juiciness; astringent	Significant differences in bitterness, bright, crunchiness, green color, green olive aroma and juiciness for LAB samples, control olives had the highest bitterness value	Randazzo et al. [105]
	Aitana, Caiazzana, Nocellara del Belice	QDA	Acid, bitter, salty; crunchiness, fibrousness, hardness; abnormal fermentation, other defects	All the tested cultivars had good sensory characteristics, and the highest scores for flesh consistency and crunchiness was obtained by Nocellara del Belice olives	Romeo et al. [106]
	Nocellara Etnea	QDA	See Pino et al. [104]	The control olives obtained the highest scores for acidity, the highest bitter taste was scored in the olives without LAB. The samples at 5% and 8% NaCl added with the LAB received the highest overall acceptability	Pino et al. [107]
LAB + Y (Natural)	Bella di Cerignola	QDA	Crunchiness; acid, bitter, salty, sweet; olive flavor, off flavor	The assessors ranked better the olives fermented with starters, with respect to the control that obtained the lowest values for crunchiness and olive flavor and the best evaluations for acid, bitter and off flavor	De Angelis et al. [108]
	Conservolea, Kalamata	QDA	Acidity, bitterness, saltiness; odor; hardness; overall	Kalamàta olives obtained the best scores for aroma and overall acceptability when the Y+LAB and MIX inoculations were used, while Conservolea olives showed the same results when LAB+Y were inoculated	Chytiri et al. [109]
Y (Natural)	Taggiasca		Acid, bitter, salty; crunchiness, fibrousness, hardness	The best combination may be obtained with the use of Y on acidified brines at the highest NaCl concentration	Ciafardini et al. [110]
LAB (treated)	Nocellara del Belice	QDA	Bright, green color; green olive aroma, off odor; crisp, easy stone, juicy; acid, bitter, salt, sweet; astringent; green olive flavor, off flavor; overall.	Data highlighted that *L. pentosus* improved the sensory characteristics of olives, with respect to control samples	Aponte et al. [111]

*LAB= lactic acid bacteria; Y=yeast.
